# Survey research on the habitation and biological information of *Callipogonrelictus* Semenov in Gwangneung forest, Korea and Ussurisky nature reserve, Russia (Coleoptera, Cerambycidae, Prioninae)

**DOI:** 10.3897/zookeys.792.26771

**Published:** 2018-10-23

**Authors:** Seung-Gyu Lee, Cheolhak Kim, Alexander V. Kuprin, Jung-Hoon Kang, Bong-Woo Lee, Seung Hwan Oh, Jongok Lim

**Affiliations:** 1 Korea National Arboretum, Pocheon 11186, South Korea Korea National Arboretum Pocheon Korea, South; 2 Osang K-insect Biological Resource Research Center, Yesan 32426, South Korea Osang K-insect Biological Resource Research Center Yesan Korea, South; 3 Federal Scientific Center of the East Asia Terrestrial Biodiversity, Far East Branch of the Russian Academy of Sciences, Vladivostok, 690022, Russia Federal Scientific Center of the East Asia Terrestrial Biodiversity, Far East Branch of the Russian Academy of Sciences Vladivostok Russia; 4 National Research Institute of Cultural Heritage, Daejeon 34122, South Korea National Research Institute of Cultural Heritage Daejeon Korea, South; 5 Korea Forest Service, Daejeon 35208, South Korea Korea Forest Service Daejeon Korea, South

**Keywords:** Coleoptera, *
Callipogon relictus
*, critically endangered species, Gwangneung forest, habitation, Korea natural monument

## Abstract

An investigation on the habitation of *Callipogonrelictus* Semenov, 1899 in Gwangneung forest was carried out, where the Korea National Arboretum is located. In an investigation spanning the last eleven years (2007–2017), 22 emergence holes, nine pupal chambers, six adults, and two larvae of *C.relictus* were identified. In this study, biological information about habitation of *C.relictus* is provided by comparing and combining the results of this investigation with a survey conducted in Ussurisky Nature Reserve, Russia, in 2015. The distribution is also reviewed to include the Korean Peninsula and a new location of South Korea is added to the distribution for *C.relictus*.

## Introduction

The genus *Callipogon* Audinet-Serville, 1832, includes nine species in five subgenera worldwide, one of which, C. (Eoxenus) relictus Semenov, 1898, is found in East Asia, while the other eight species are mainly distributed in Central and South America, including Mexico, Guatemala, and Colombia (Lameere 1904; Monne 2017). The presence of *C.relictus* in East Asia may provide evidence that the old world and the new world were connected when the Bering land bridge was above sea level (Kuprin and Bezborodov 2012).

*Callipogonrelictus*, which is known to be one of the largest Coleoptera species in the Palearctic region, was first recorded in Vladivostok, far eastern Russia, and is also found in some parts of China, Mongolia and central and northern parts of the Korean Peninsula (Byun et al. 2007; Li et al. 2013; Yi et al. 2018).

**Figure 1.**
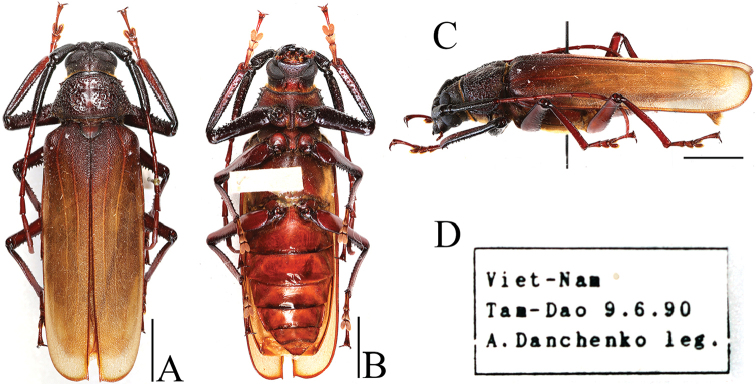
Macrotoma (Bander) fischeri, male: **A** dorsal aspect **B** ventral aspect **C** lateral aspect **D** label data. Scale bars: 10 mm.

In the first report from the Korean Peninsula, *Callipogonrelictus* was misidentified by Saito (1932) as *Macrotomafisheri* Waterhouse, 1884 (Figure 1) with no precise collection record. Later, Cho (1934) collected specimens from Mt. Bukhansan in Seoul, the capital of South Korea, and additionally summarized the host plants and the distribution in 1955. Murayama (1936) first recorded the appearance of larvae collected from Gwangneung forest in Pocheon-si, Gyeonggi province, and reported that *Carpinuslaxiflora* (Siebold & Zucc.) Blume (Betulaceae) was its host plant. Thereafter, when a large number of specimens were collected from Chujeon-ri, Buksan-myeon, Chuncheon-si, Gangwon province, this region was designated as a natural monument No. 75 on 3 December 1962, a habitat of *C.relictus*. However, the habitat was destroyed during construction of the Soyanggang dam, and its natural monument status was annulled on 19 July, 1973.

Detailed and quantitative investigations of the Korean distribution and population size of *Callipogonrelictus* have not been conducted, but the population density has been observed to be decreasing rapidly, and for conservation, the species was afforded legal protection, designated as a natural monument No. 218, on 20 November 1968, by the Korean Cultural Heritage Administration. It was designated a class I endangered species by the Ministry of Environment on 31 May 2012, and was declared to be “Critically Endangered” (CR) on 6 December 2013 (Cho and Lee 2013).

*Callipogonrelictus* is being protected as a CR species based on its dwindling numbers both in Korea and in its type locality, Russia. However, due to the difficulty of obtaining specimens, there has been very little ecological research on the species.

Several Korean researchers have conducted investigations of habitation, taxonomical studies on, and research into the measures of conservation of *Callipogonrelictus*. In particular, Kim et al. (1976) surveyed the state of habitation in Yangju-gun, Gyeonggi province (currently Gwangneung forest, Pocheon-si) and Gangneung-si, Gangwon province (currently Sogeumgang, Gangneung-si), and recorded the occurrence frequency, distribution, and host plants of adults, and the appearance of larvae. Hong and Kim (1991) discussed the distribution, morphological variation in adult and biological information based on 28 Korean specimens. Byun et al. (2007) surveyed habitation of *C.relictus* in Gwangneung forest between 1999 and 2006 and discussed various on-site conservation measures. An (2010) reviewed the Korean literature and proposed measures for the systematic conservation and management of *C.relictus*. Lim et al. (2013) performed a molecular study of a large larva found in Gwangneung forest in 2010 and on an adult of *C.relictus* collected in 1990. They identified that the specimens belonged to the same species, while also re-describing the morphology of the larva. Lim et al. (2017) used a female adult discovered in Gwangneung forest in 2014 and 2015 to conduct mitochondrial genome analysis. Recently, various research institutes in the East Asia, including the Korea National Arboretum, have taken an interest in *C.relictus*, and through international collaboration, are engaging in diverse research to establish conservation measures. However, there is a severe lack of basic biological data on *C.relictus*, making it very difficult to collect specimens, and, owing to the rapid decline in its population size, they are rarely observed lately.

In this study, we combined the results of investigations that we conducted between 2007 and 2017 in Gwangneung forest (currently, the only known habitat in Korea) and we compared the results with a habitation survey in the Ussurisky nature reserve in Russia, type locality and one of the largest habitat of this species. Finally, we provide biological information that can aid species conservation measures by reviewing all reported information of *C.relictus* previously discovered in Gwangneung forest and in the rest of Korea, as well as various researches in other countries.

## Materials and methods

**Figure 2.**
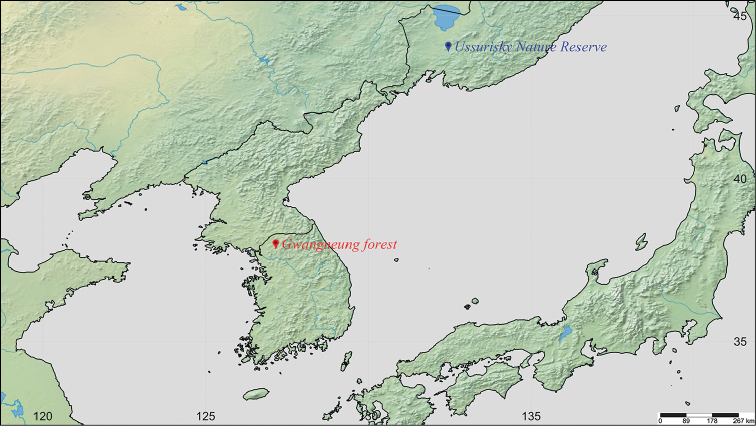
Locations of investigation sites (red symbol: Gwangneung forest, Korea; blue symbol Ussurisky nature reserve, Russia).

The major investigation area was the whole Gwangneung forest, spanning Namyangju-si and Pocheon-si in Gyeonggi province (Figure 2, red). In particular, the investigation focused on the northeast part of Soribong, where *Callipogonrelictus* have often been collected in the past.

Another investigation area was the Ussurisky nature reserve, Primorsky Krai, Russia (Figure 2, blue) with the cooperation of Dr. Alexander Kuprin (Far East Branch, Russian Academy of Sciences). We conducted an investigation focusing on two areas with a high density of *Callipogonrelictus* habitation (43°37'58.5"N, 123°13'57.0"E, Alt. 78; 43°39'49.7"N, 132°30'1.73"E, Altitude 164 m).Survey research on the habitat in Gwangneung forest, Korea, was conducted over 11 years, from 2007 to 2017. Between 2007 and 2009, the research was conducted once a month from June to August, when adults had previously been found; between 2010 and 2016, surveys were conducted once a month from March to June, and at least twice a month from July to August. In 2017, surveys were conducted once a week from February to May and in September, and twice a week from June to August. At the Ussurisky nature reserve (Russia), one survey was conducted from 25–27 August 2015.

In order to survey research on the habitat in the immature stage, we first looked for the presence of adult emergence holes, focusing on dead trees, which have been reported to be host plants, and measured the size, height, direction of the holes and the diameter at breast height (DBH) of the tree. When emergence holes were found, they were opened to check for the presence of larvae and to measure the size of any feeding scars and pupal chambers.

**Figure 3.**
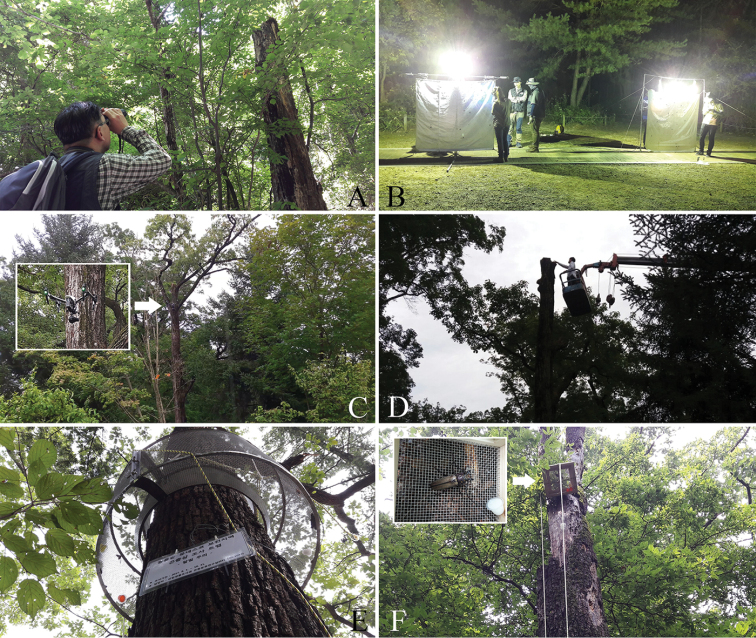
Investigation methods: **A** visual survey (naked eye) **B** light trap **C** heli-cam **D** ladder truck **E** bait trap **F** pheromone trap.

In order to survey adults directly, during the day we conducted a visual survey using binoculars to look for the appearance of adults near the sap of the host plants (Figure 3A); at night, we used a light trap to try to lure adults (Figure 3B). When necessary, a heli-cam (helicopter camera) and a ladder truck were used to look for emergence holes in high places and trap surveys were conducted using other types of trap, including bait trap with rotting fruit and sap fermented with honey, circular cage traps, and pheromone trap (Figure 3C–F).

## Results

### Occurrence frequency of adult

**Figure 4.**
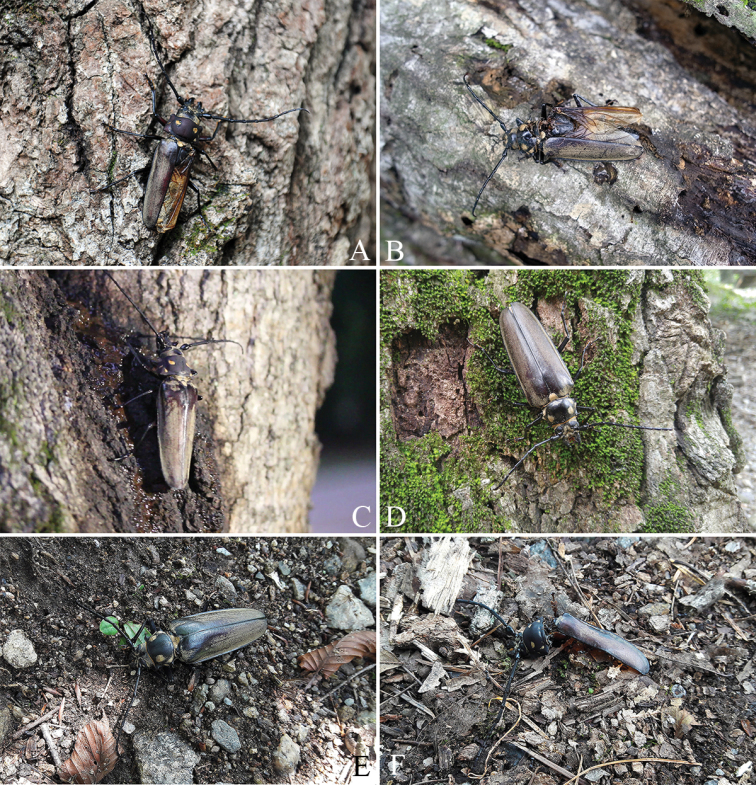
Adults of *Callipogonrelictus* discovered in Gwangneung forest: **A** male in 2014 **B** female in 2015 **C** male in 2016 **D** female in 2017 **E** female in 2017 **F** male in 2017.

During the research period (2007–2017), we observed a total of six adults during four consecutive years: one male in 2014, one female in 2015, one male in 2016, and one male and two females in 2017 (Figure 4A–F, Tables 1–2).

The body length (from apex of mandible to apex of elytra) of the male found on 19 August 2014 was 88.0 mm (Figure 4A). At the time of finding, the right elytron was missing, and much of the left elytral pubescence was missing. The individual was in a state of exhaustion and died the next day.

The body length of the female found on 27 July 2015 was 78.0 mm (Figure 4B). At the time of its finding the pronotum was severely damaged, the left elytron was missing, and much of the right elytral pubescence was missing. The mesonotum was partially detached and the individual was gathered by numerous ants, dying within a few hours of discovery.

The body length of the male found on 10 August 2016 was 98.0 mm (Figure 4C). At the time of finding, there was a crack running longitudinally in its pronotum, much of the pubescence was missing, and some claws were also missing. The individual was in a state of exhaustion and died the next day.

The body length of the female found on 20 July 2017 was 78.0 mm (Figure 4D). At the time of finding, there were no particularly damaged parts; some of the pubescence was missing, but the insect was moving actively. After artificial egg collection, the individual died on 31 July 2017.

The body length of the female found on 11 August 2017 was 75.6 mm (Figure 4E). At the time of finding, its right tibiae and tarsi were missing, much of the pubescence was missing, and the ovipositor was prolapsed. The individual was in a state of exhaustion and died in under an hour.

The body length of the male found on 14 August 2017 was 60.0 mm, making it the smallest of all the males discovered to date (Figure 4F). At the time of discovery, the individual was already dead; the left elytron, some legs, and the whole abdomen were missing, and there were ants gathering on the inside of the pronotum.

### Emergence holes

**Figure 5.**
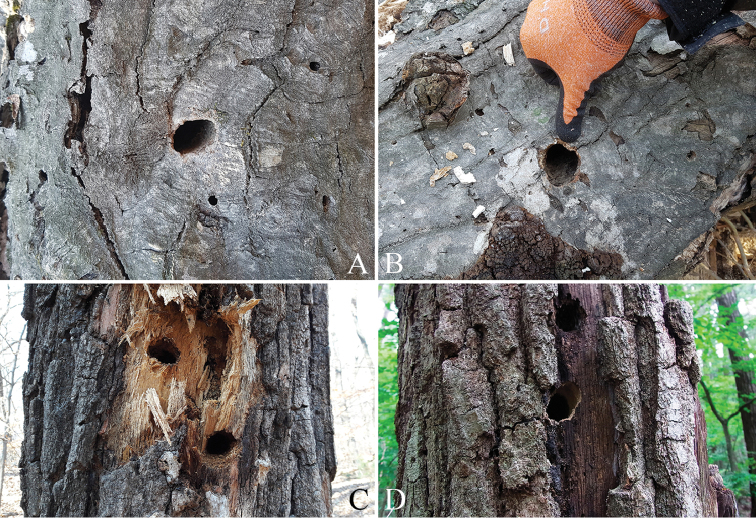
Emergence hole of *Callipogonrelictus* in Gwangneung forest, Korea: **A–B** emergence hole on *Carpinuslaxiflora***C** emergence hole on *Quercus* sp. **D** emergence hole on *Quercusaliena*.

In the survey in Gwangneung forest, we found a total of 22 emergence holes on four species of dead tree (*Carpinuscordata* Blume, *C.laxiflora* (Siebold & Zucc.) Blume, *Quercusaliena* Blume, and *Q.mongolica* Fisch). The largest number of holes was found on *C.laxiflora* and as many as six holes were found on a single tree. The holes were observed at heights of 0.9–3.1 m, and six holes faced north and five holes faced northeast (Table 3). The shapes of the emergence holes were subcircular to elliptical, with most holes having a slanted elliptical shape; the mean width and height were 32.1 mm and 26.1 mm, respectively (Figure 5A–D, Table 4).

**Figure 6.**
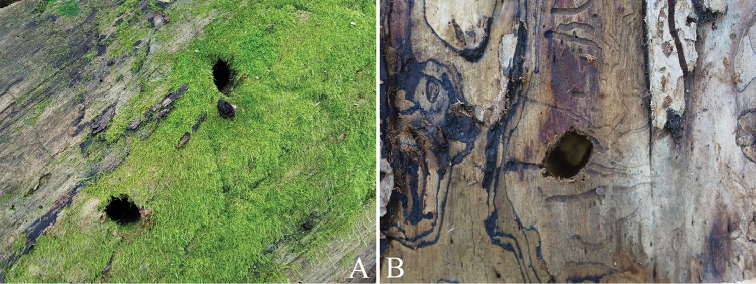
Emergence hole of *Callipogonrelictus* in Ussurisky nature reserve, Russia: **A–B** emergence hole on Ulmusdavidianavar.japonica.

In Ussurisky nature reserve, we observed a total of 56 emergence holes, on Ulmusdavidianavar.japonica (Rehder) Nakai only. The DBH of the dead trees with emergence holes was 56–130 cm, and there were as many as 18 holes on the only one tree (that could be observed with naked eyes). The emergence holes were found at various heights in the range of 1.0–24.0 m (Table 3). The shapes of the holes were very similar to those found in Korea, albeit a little larger, with a mean width and height of 33.4 mm and 28.2 mm, respectively (Figure 6A–B, Table 4).

### Larvae and feeding scars

**Figure 7.**
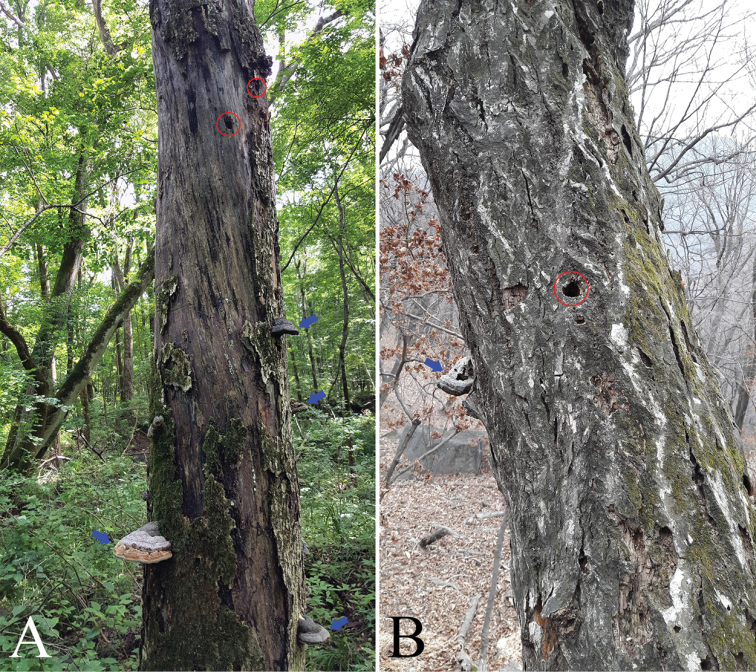
Emergence hole and fungus on host plant (red circle, emergence hole; blue arrow, fungus): **A**Ulmusdavidianavar.japonica**B***Carpinuscordata*.

**Figure 8.**
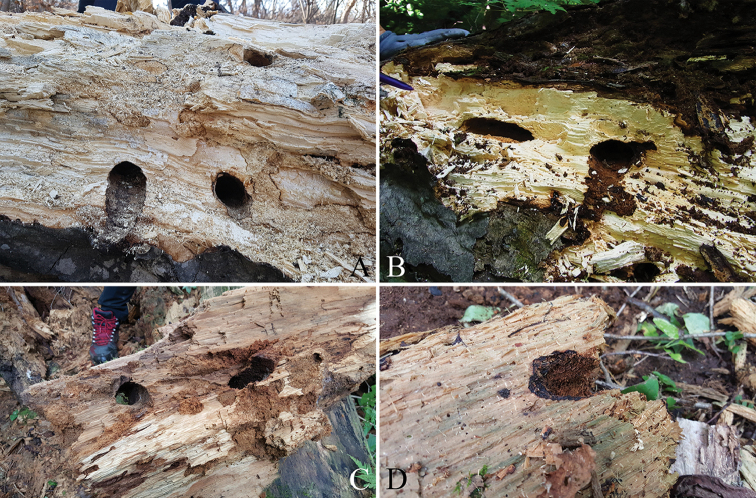
Larval gallery and pupal chamber of *Callipogonrelictus* in Gwangneung forest.

Larval feeding scars of *C.relictus* were mostly observed on old (DBH: ≥ 35 cm), less vigorous *Carpinus* spp. and *Quercus* spp., and were observed more commonly on standing dead trees than on fallen trees. Most of the trees with feeding scars have fungal growth (Figure 7A, B) or fungal infection around or on the surface of the feeding scars (Figure 8A, B).

**Figure 9.**
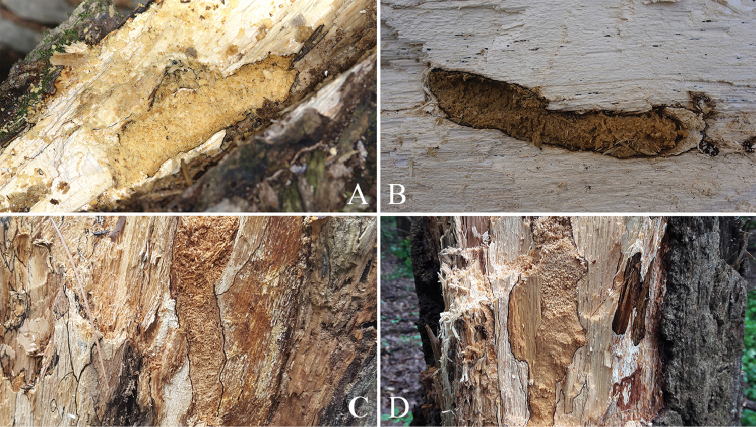
Larval excreta of *Callipogonrelictus* in gallery: **A–B** larval excreta in *Carpinuslaxiflora***C–D** larval extra in *Quercusaliena*.

The feeding scar tunnels stretched from below the bark to deep within the trunk. In general, the tunnels had a gradually widening shape oriented vertically in relation to the ground, but the shapes were highly irregular. Some tunnels were horizontal to the ground, and sometimes vertical and horizontal tunnels intersected. Larval excreta varied according to the type and maturity of the host plant and according to the age of the larvae but they were typically slightly thicker than those of the other cerambycids and similar to those of *Dorcushopeibinodulosus* Waterhouse, 1874 (Coleoptera: Lucanidae) (Figure 9A–D).

**Figure 10.**
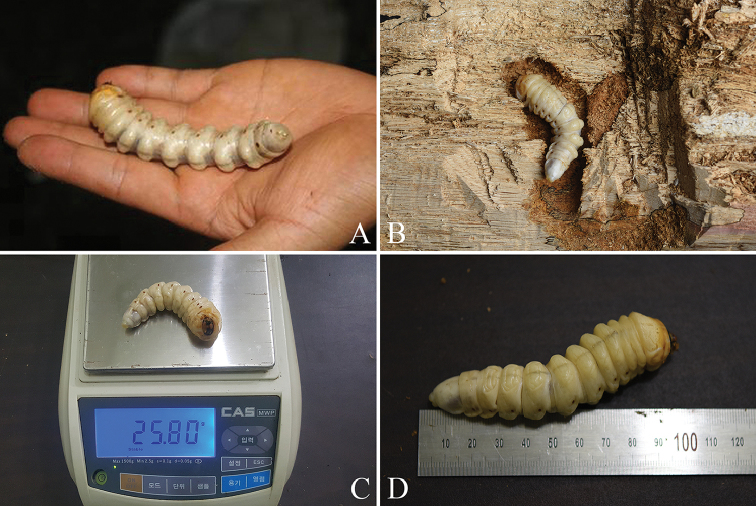
Larvae of *Callipogonrelictus* discovered in Gwangneung forest: **A** larva in 2010 **B–D** larva in 2016.

Two larvae were found, one in 2010 and one in 2016. The larva collected in a *C.laxiflora* tree on 29 July, 2010 had a length of 11.6 cm, a head width of 1.14 cm, and was found dead, infected with a pathogenic organism (Figure 10A). The larva discovered in a *Q.aliena* tree on 1 August, 2016 was very healthy, with a length of 10.5 cm, a head width of 1.14 cm, and a weight of 25.8 g (Figure 10B–D).

**Figure 11.**
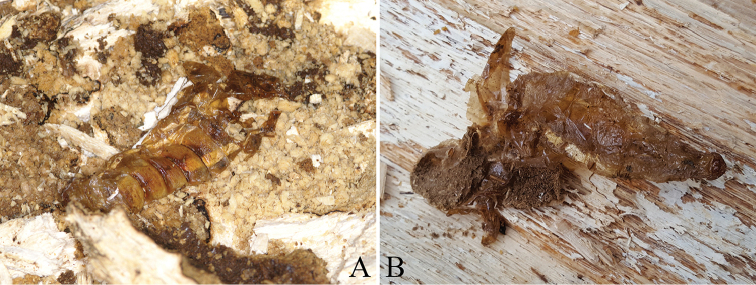
Pupal exuvium of *Callipogonrelictus* in Gwangneung forest: **A** exuvium in *Carpinuslaxiflora* in 2012 **B** exuvium in *Quercusaliena* in 2016.

Two larval exuviae were collected, one in 2012 and one in 2016. One pupal exuvia was found in a *C.laxiflora* tree on 26 July 2012 (Figure 11A) and one female pupal exuvia was found in the same *Q.aliena* of dead tree where the larva was discovered on 1 August, 2016 (Figure 11B). In addition, in 2017, the right mandible of a larva was found in a dead *C.laxiflora* tree.

### Pupal chambers

**Figure 12.**
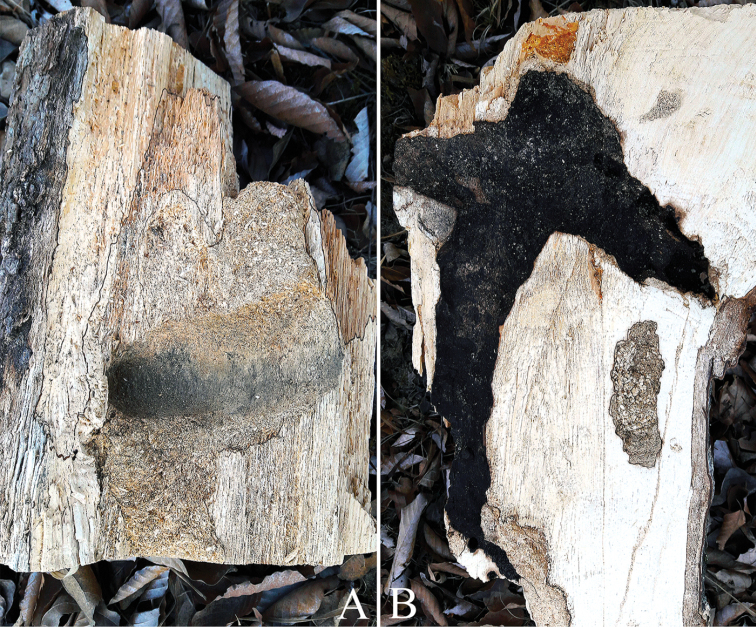
Pupal chambers of *Callipogonrelictus* in Gwangneung forest: **A** pupal chamber in *Carpinuslaxiflora***B** pupal chamber in *Quercusaliena*.

During the study period (2007–2017), we found a total of nine pupal chambers of *C.relictus* in three species of dead trees in Gwangneung forest (*Carpinuscordata*, *C.laxiflora*, and *Q.aliena*). The highest number of pupal chambers was found in *C.laxiflora* trees and as many as three pupal chambers were found in one *C.laxiflora* tree. The DBH of dead trees containing pupal chambers was in the range 39–69 cm, with a mean DBH of 57.1 cm. The pupal chambers were either ellipses with a long horizontal axis, or long tunnel shapes. They were 13–20 cm in length, located 4.5–10.0 cm deep inside the bark, parallel to the ground and perpendicular to the tree trunk (Figure 12A, B, Table 5).

### Host plants and habitats

In the Gwangneung forest in Korea, direct identification of individuals and signs of habitation occurred in four species of tree: *Carpinuscordata*, *C.laxiflora*, *Quercusaliena*, and *Q.mongolica* (Figs 5A–D, 7B). In addition, we observed a number of feeding scars in other *Quercus* spp. that could not be precisely identified at the species level, due to the high extent of decay in these dead trees. Most of these trees were located in areas on the northern slope, close to the valley, where the terrain was not too steep.

**Figure 13.**
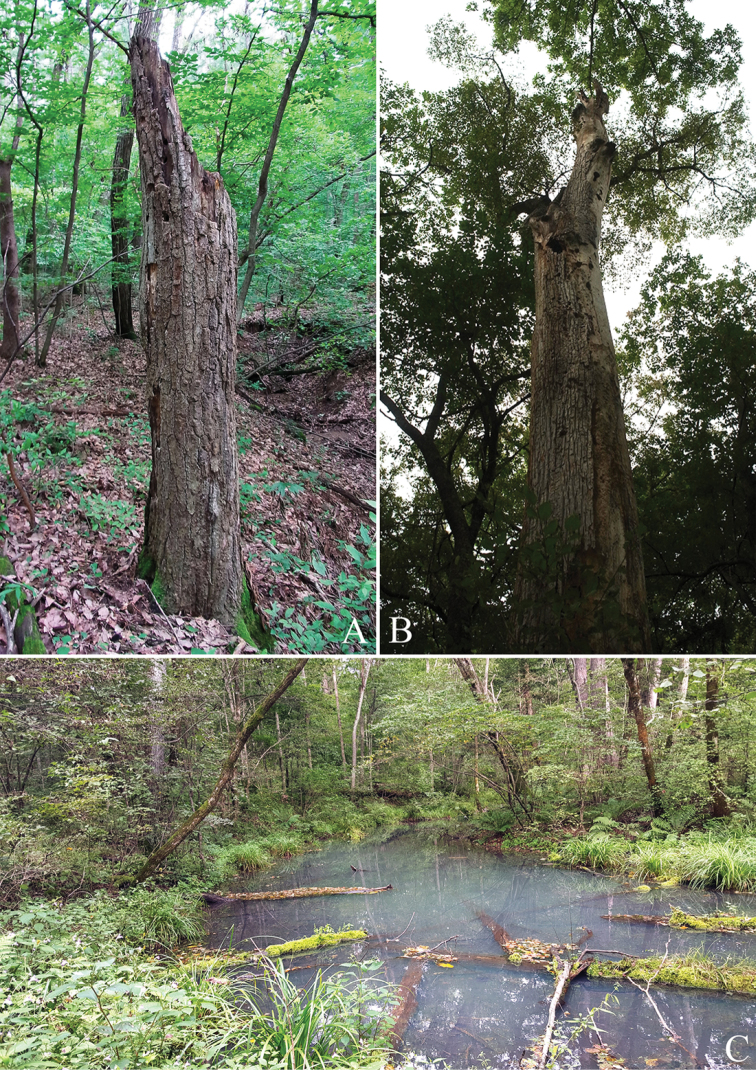
Host plants and habitat of *Callipogonrelictus*: **A***Quercusaliena* in Gwangneung forest **B**Ulmusdavidianavar.japonica in Ussurisky nature reserve **C** forest in Ussurisky nature reserve.

The species in which we directly observed larvae were *C.laxiflora* and *Q.aliena*. The state of decay of *Q.aliena* was as follows: trunk left standing; approximately 80–90% bark remaining; bark tightness tight to loose; wood texture hard; shape of cross section round; branches absent (Figure 13A).

At the Ussurisky nature reserve in Russia, we identified various host plants such as *Fraxinusmandschurica*, *Quercusmongolica*, *Ulmuslaciniata*, and U.davidianavar.japonica – but emergence holes of *C.relictus* and feeding marks were observed only in U.davidianavar.japonica (Figure 13B); overall, this region contains several tributaries and the mountain wetlands are well developed (Figure 13C).

## Discussion

### Distribution in the Northeast Asia

*Callipogonrelictus* has so far been reported from four countries in East Asia: Russian Far East, China and Korean Peninsula (Kuprin and Bezborodov 2012; Bezborodov 2016; Yi et al. 2018).

In the Russian Far East, which is type locality of this species, its distribution was summarized by Kuprin and Bezborodov (2012). They reported that *C.relictus* were distributed across four federal subjects of Russia (Primorsky Krai, Khabarovsk Krai, the Jewish Autonomous Oblast, and Amura Oblast) and distinguished four local populations: Ussuriiskaya, Khoro-Bikinskaya, Khingano-Bure-inskaya, and Selemdzhinskaya populations (Kuprin and Bezborodov 2012).

The distribution of *C.relictus* in China has been reported to span eight provinces: Tianjin, Gansu, Hebei, Heilongjiang, Inner Mongolia, Jilin, Liaoning, Shaanxi, and Shanxi (Li et al. 2014). Of these, the largest number of individuals has been recorded in the three provinces (Heilongjiang, Jilin, and Liaoning) that border North Korea and Primorsky Krai, the type locality in Russia (Bezborodov 2016).

Recently, Bezborodov (2016) and Yi et al. (2018) reassessed the distribution of *C.relictus* based on sampling data. Based on current reports, the eastern limit of the distribution of *C.relictus* is the Imeni Lazo district, Khabarovsk Krai, Russia, located at 135°E (Kuprin and Bezborodov 2012); the northern limit is the Selemdzhinsky district, Amur Oblast, Russia, located at 53°N; the southwestern limit is Houyugou city, Shaanxi, China, located at 35°N, 110°E (Hua 2002); the northeastern limit is Mt. Si-fang-shan in A-er-jin city, Inner Mongolia, China, located at 49°N, 123°E (Li et al. 2013); and the southeastern limit is Sogeumgang of Mt. Odaesan, Gangwon province, South Korea, located at 37°N, 128°E (Yi et al. 2018). So far, *C.relictus* has been observed in the range of latitudes 35°N to 53°N and longitudes 110°E to 135°E. It is possible that the known range will expand if more precise surveys are conducted in Northeast Asia.

### Distribution in the Korean Peninsula

The first record of *Callipogonrelictus* in South Korea was provided by Saito (1932), who misidentified the species as *Macrotomafisheri*, after which Cho (1934) collected and reported *C.relictus* at Namjangdae in Mt. Bukhansan, Seoul.

**Figure 14.**
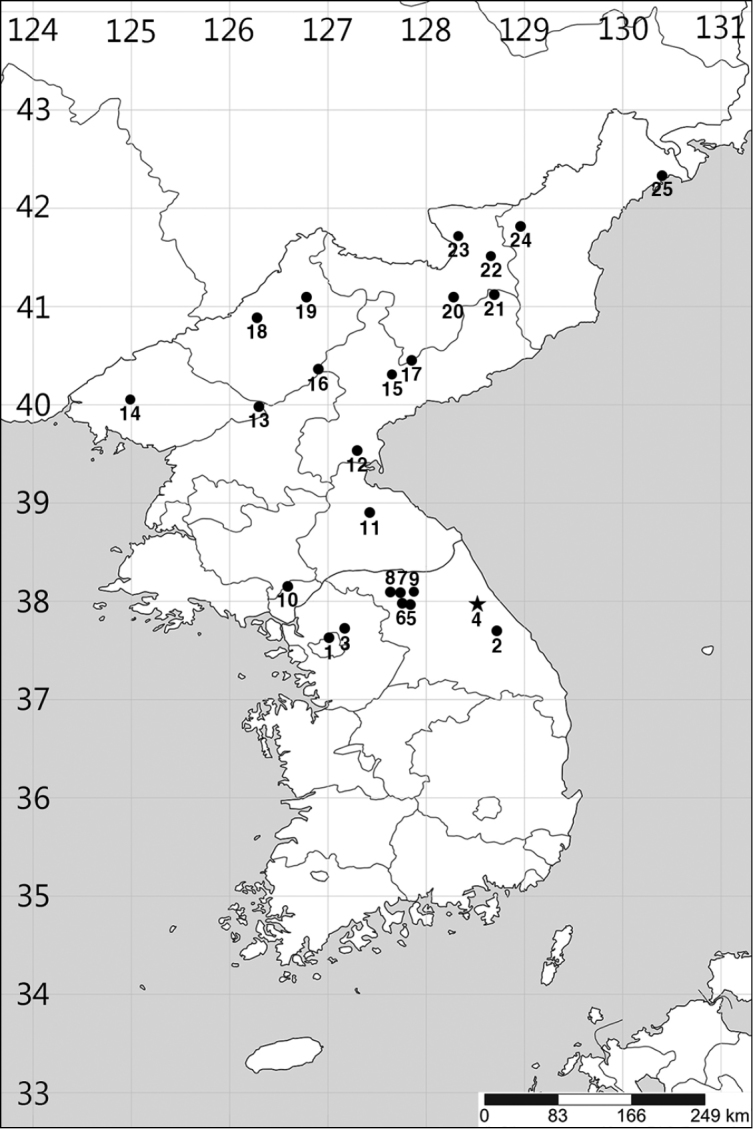
Distribution map of *Callipogonrelictus* in the Korean Peninsula (An 2010; Bezborodov 2016; Yi et al. 2018; *per. obs.*): **1** Gyeonggi Province , Seoul-si, Mt. Bukhansan, Namjangdae **2** Gangwon Province, Gangneung-si, Mt. Odaesan, Sogeumgang **3** Gyeonggi Province, Pocheon-si, Gwangneung forest **4** Gangwon Province, Yangyang-gun, Seo-myeon (new record) **5** Gangwon Province, Chuncheon-si, Buksan-myeon, Chujeon-ri **6** Gangwon Province, Chuncheon-si, Buksan-myeon, Cheongpyeong-ri **7** Gangwon Province, Hwacheon-gun, Gandong-myeon, Yuchon-ri **8** Gangwon Province, Hwacheon-gun, Hanam-myeon, Georye-ri **9** Gangwon Province, Yanggu-gun, Yanggu-eup **10** Hwanghaebuk Province, upper region of the Imjingang River **11** Gangwon Province, Sepo-gun, Sambang-ri (Chuga-ryeong, Sambang-hyeop) **12** Hamgyeongnam Province, Geumya-gun **13** Pyeonganbuk Province, Mt. Myohyangsan **14** Pyeonganbuk Province, Mt. Cheonmasan **15** Hamgyeongnam Province, Sinheung-gun **16** Yanggang Province, Mt. Nangnimsan **17** Hamgyeongnam Province, Mt. Bulgaemiryeong **18** Jagang Province, Mt. Baekamsan **19** Jagang Province, Mt. Hwangsuryeong **20** Yanggang Province, Gapsan-gun **21** Hamgyeongnam Province, Mt. Duryubong **22** Jagang Province, Baekam-gun **23** Jagang Province, Mt. Bukpotaesan **24** Hamgyeongbuk Province, Yeonsa-gun **25** Hamgyeongbuk, Seonbong-gun.

Cho (1955) added a record of distribution in two regions in Gyeonggi province (Pocheon-gun and Paju-si) and four regions in Gangwon province (Chuncheon-si, Hwacheon-gun, Yanggu-gun, and Gangneung-si) in the north of South Korea. In addition, Cho (1961) reported the following specific locations from where specimens were collected: Chujeon-ri (Buksan-myeon, Chuncheon-si), Georye-ri (Hanam-myeon, Yuchon-ri, Gandong-myeon, Hwacheon-gun), Eupnae-ri (Yanggu-myeon, Yanggu-gun) (Gangwon province), Bukhan-san (Seoul) and Gwangneung (Gyeonggi province) (Figure 14).

Kim (1978) reviewed specimens of *C.relictus* deposited in various Korean research institutions and found that, after the addition of records for Gangwon province (Cheongpyeong-ri, Buksan-myeon, Chuncheon-si, Sogeumgang, Odae-san, and Gangneung-si), the only official collection records came from Gwangneung forest in Pocheon-si, Gyeonggi province (Byun et al. 2007: Table 2).

**Figure 15.**
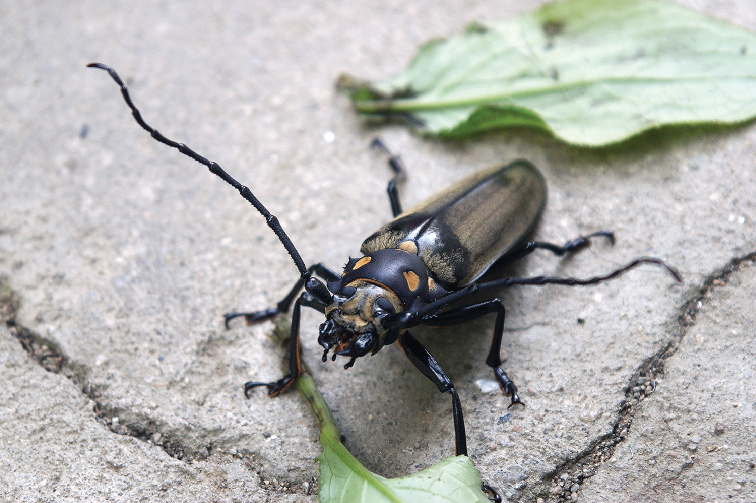
Adult male of *Callipogonrelictus* in Yangyang-gun of Gangwon province (photograph taken by Jeong-Ok Kim)

In this study, we found adults of *C.relictus* in the Gwangneung forest for four consecutive years from 2014 to 2017 (Table 1) and we also added a new location to the Korean distribution of the species in Yangyang-gun, Gangwon province, based on photographic evidence (Figure 15). This region is thought to be a suitable alternative conservation site (habitat), as it has not only a much wider habitat area than Gwangneung forest and little human interference, but also a large population of host plants. Habitation of Korean individuals has also been verified recently in this region.

In North Korea, *C.relictus* was first recorded by Nakane (1974) and up to now it has been reported to have a wide distribution in the following provinces: Gangwon province, Yanggang province, Jagang province, Pyeonganbuk province, Hamgyeongnam province, Hamgyeongbuk province, and Hwanghaebuk province (Bezborodov 2016; Yi et al. 2018). Of these, the largest numbers of individuals were reported in Yanggang province, Jagang province, Pyeonganbuk province and Hamgyeong province, which are adjacent to Russian Primorsky Krai and China northeastern regions (Jilin, Liaoning), with as many as 31 individuals being collected in a single survey (Mt. Bukpotaesan, Yanggang province) (Yi et al. 2018), suggesting a stable population inhabiting the area.

**Table 1.** Signs of habitation of Callipogon (Eoxenus) relictus discovered in Gwangneung forest in 2007–2016 (units: ex).

	2007	2008	2009	2010	2011	2012	2013	2014	2015	2016	2017	Total
**Larva**	–	–	–	1	–	–	–	–	–	1	–	**2**
**Pupal chamber**	–	–	–	2	2	2	1	–	–	1	–	**8**
**Exuvium**	–	–	–	–	–	1	–	–	–	1	–	**2**
**Adult**	–	–	–	–	–	–	–	1 ♂	1♀	1♂	1♂ 2♀	**6**
**Emergence hole**	–	–	–	3	2	3	2	2	–	1	–	**13**
**Total**	–	–	–	6	4	6	3	3	1	5	3	**31**

### Survey research on habitation of *Callipogonrelictus* in Gwangneung forest

Our study recognized that, except for the new record from Yangyang-gun (Gangwon province), Gwangneung forest is the only collecting site of *C.relictus*. Kim et al. (1976) performed 20 surveys between June and September when adults of *C.relictus* occur, but they did not observe the adults, indicating that the population density was already very low at that time.

During our study, through focused surveys on habitation, we directly found six adults, two larvae, and various other signs of *C.relictus* (22 emergence holes, nine pupal chambers, tunnels, and feeding scars) (Table 1), providing that the Korean population, although low in number, is still persisting in Gwangneung forest and is not extinct in the South Korea.

### Conditions of adults

Of the six adults discovered in this study, except one individual, all five were either exhausted from severe damage and died within a day, or were already dead. Three of which individuals were missing an elytron (Figure 4A–B, F) and another was found with a crack in the pronotum (Figure 4C); these damages are considered to be the result of attacks by natural enemies (woodpeckers, squirrels, wild animal, etc.)

In previous studies, adults were observed in Gwangneung forest between June and September and particularly, high numbers were observed in August (Byun et al. 2007: Table 3). Similarly, in our study, we found two adults in July and four adults in August, meaning that the majority of adults were observed in August, consistent with the findings of other studies (Table 2). However, the individuals that we discovered in August were severely weakened, to the extent that they could not cling to a tree or were already dead. Given that they were also missing much of their pubescence, we expect that these individuals were also active as adults in July.

During this study, we discovered a total of six adults in Gwangneung forest but, apart from a male that flew over from a nearby factory, we found the other five adults in regions with a high density of *Quercus* spp. with flowing sap. Like other Prioninae species, *C.relictus* adult is known to be attracted by light at night (Lee 1987; Byun 2007). The male discovered at a factory in 2016 was also thought to have been attracted by bright light at nighttime, while the other five individuals were probably found during egg-laying or feeding activity.

We succeeded in collecting 16 eggs from a female found under a *Quercus* sp. tree through artificially induced oviposition; the hatched larvae are being reared to increase their population. Given that we consistently found a small number of adults over the last four years, although the population size is small, the population of *C.relictus* appears to remain more stable here than in the other parts of Korea.

**Table 2.** Larvae of Callipogon (Eoxenus) relictus discovered in Gwangneung forest in 2007–2017.

Year	Month		Collecting site
	July	August	
2014	–	19^th^ (♂)	Korea National Arboretum
2015	27^th^ (♀)	–	Korea National Arboretum
2016	–	10^th^ (♂)	near Mt. Jukyeopsan
2017	20^th^ (♀)	11^th^ (♀), 14^th^ (♂)	Korea National Arboretum

**Table 3.** Characteristics of emergence holes of Callipogon (Eoxenus) relictus identified in Gwangneung forest and the Ussurisky Nature Reserve (*CC, *Carpinuscordata*; CL, *C.laxiflora*; QA, *Quercusaliena*; QM, *Q.mongolica*; Qsp, *Quercus* sp.; UJ, Ulmusdavidianavar.japonica; NO, North; NE, Northeast).

	Gwangneung forest (Korea)												Ussurisky Nature Reserve (Russia)						
	1	2	3	4	5	6	7	8	9	10	11	12	1	2	3	4	5	6	7
**Host plant**	CL	CL	CL	CL	CL	CL	CC	QM	QA	Qsp	CL	CL	UJ	UJ	UJ	UJ	UJ	UJ	UJ
**Diameter of breast height (cm)**	69	60	59	55	50	53	48	46	36	51	72	–	56	125	130	70	60	110	80
**Number of emergence hole**	2	2	2	1	1	2	1	1	1	2	1	6	1	5	18	13	6	1	12
**Height of emergence hole (m)**	0.9/ 1.2	1.6/ 3.1	2.2/ 2.6	2.2	2.4	2.1/ 3.1	1.8	1.9	2.1	1.2/ 1.3	3.1	–	1.8	1.8–2.3	1.0–24.0	1.0–4.0	2.0–12.0	1.5	2.0–10.0
**Direction of emergence hole**	NO	NO	NE	NO	NE	NO	NE	NO	NO	NE	NE	–	–	–	–	–	–	–	–

### Conditions of immature stages

The signs of habitation by immature stages of *C.relictus* (emergence holes, pupal chambers, tunnels, and feeding scars) were mostly found on *Carpinus* spp. and *Quercus* spp. of dead trees with DBH ≥ 39 cm, in forest areas where *C.laxiflora* were dominant and there were a mixture of *Quercus* spp. Because fallen trees have a large surface in contact with the ground, they decay rapidly and there is secondary invasion by wood boring insects (e.g. Lucanidae, Tenebrionidae). It was difficult to distinguish the signs of habitation of *C.relictus* from those of other species. In the natural state, the immature period is very long, over 4–5 years (Li et al. 2012), and so, rapidly decaying dead trees are predicted to be disadvantageous for survival.

The shapes of the emergence holes we found were mostly elliptical, but some were close to being circular (Figs 5A–D, 6A–B). Differences in the hole shape have been reported to be correlated with mandible length (Yi 2014), but this has still not been clearly elucidated. In this study, the majority of emergence holes were elliptical, consistent with that of other prognathous species in the subfamily Prioninae (e.g. *Prionusinsularis* Motschulsky and *Aegosomasinicum* White), suggesting that differences arise according to structure of head and mouthparts (prognathous, hypognathous, or retrognathic) rather than mandible length. The size of the holes was clearly larger than that of other large other Korean cerambycids (e.g. *Batoceralineolata* Chevrolat; *Neocerambyxraddei* Blessig & Solsky; *A.sinicum* White) and this is considered to be related to the body width (Korean averages: width 32.1 mm, length 26.0 mm; Russian averages: width 34.6 mm, length 28.2 mm). We found that the emergence holes have towards the north or northeast, and this is thought to be a strategy to minimize moisture loss due to excessive sunlight.

Pupal chambers were most commonly observed in *C.laxiflora*, with lengths in the range of 13–20 cm (Figure 12A, B). The length and size of the pupal chambers was correlated with the DBH of the host plant, where a larger DBH was associated with larger and longer pupal chambers (Table 5). This is because, in host plants with a larger DBH, there is more space available to make the pupal chamber as well as sufficient feeding resources for a favorable nutritional state, and therefore, the body can grow larger (Karino et al. 2004). We assume that the size of the pupal chamber increases in proportion with the size of the body.

The depth of the pupal chambers from the bark varied according to the level of decay and the hardness of the wood. In dead trees that were highly decayed and softer, pupal chambers were deeper; conversely, in dead trees that had harder wood, or that were less decayed, pupal chambers were shallower (Table 5). This suggests that the depth from the bark is adjusted to account for the properties of the wood, in order to facilitate emerge after exuviation.

**Table 4.** Sizes of emergence holes of Callipogon (Eoxenus) relictus identified in Gwangneung forest and the Ussurisky Nature Reserve (units: mm).

	Gwangneung forest (Korea)		Ussurisky Nature Reserve (Russia)	
	Width	Height	Width	Height
**1**	35	32	26	18
**2**	32	28	34	25
**3**	36	28	37	29
**4**	27	24	34	27
**5**	28	27	26	20
**6**	30	22	36	32
**7**	31	24	32	28
**8**	31	25	31	24
**9**	35	24	31	25
**10**	35	29	35	30
**11**	36	28	37	34
**12**	32	25	40	26
**13**	31	22	33	26
**14**	31	27	36	30
**15**	–	–	35	33
**16**	–	–	45	34
**17**	–	–	22	41
**18**	–	–	32	26
**Mean**	**32.1**	**26.1**	**33.4**	**28.2**

**Table 5.** Sizes of pupal chambers of Callipogon (Eoxenus) relictus identified in Gwangneung forest (Korea) (*CC, *Carpinuscordata*; CL, *C.laxiflora*; QA, *Q.aliena*) (units: cm).

	1	2	3	4	5	6	7	8	9	Mean
**Length of chamber**	18	20	20	15	17	18	18	14	13	**17.5**
**Longest axis pupal chamber diameter**	4.1	4.4	4.3	3.8	3.9	3.8	4.2	4.4	4.3	**4.1**
**Shortest axis pupal chamber diameter**	3.3	3.6	3.5	3.2	3.2	3.3	3.4	3.2	3.1	**3.3**
**Host plant**	CL	CL	CL	CL	CL	CL	CL	CC	QA	**3 spp.**
**Diameter at breast height**	60	69	69	55	53	50	60	59	39	**57.1**
**Depth under bark**	7.0	9.0	8.0	7.0	8.0	5.0	10.0	6.0	4.5	**7.2**

### Comparison of habitats (Gwangneung forest and Ussurisky nature reserve)

Gwangneung forest, South Korea, is located in the center of the Korean Peninsula (latitude 37°42'36"–37°47'41"N, longitude 127°8'20"–127°11'58"E), with a total area of 2,400 ha and an altitude of 40–620 m above sea level (Cho et al. 2007) (Figure 2A). The average annual rainfall is 1,433.8 mm and the average annual temperature is 11.7 °C (Korea National Arboretum 2015). Gwangneung forest not only contains approximately 20% of the recorded plant species in South Korea (Korea National Arboretum 2004) but is also reported to contain 3,794 species of insect, representing 28% of all Korean insect species (Korea National Arboretum 2006).

Managed by the Korea National Arboretum, Gwangneung forest is composed of 54% natural forest and 42% artificial forest. The natural forest consists mostly of broadleaf trees centered on Soribong, including *Carpinuslaxiflora*, *Quercusmongolica* and *Q.serrata*, while the artificial forest, mostly on low-lying ground, consists of a needleaved plantation, *Abiesholophylla*, *Pinusrigida*, and *Larixkaempferi*, and some planted broadleaved trees (Korea National Arboretum 2015).

In this study, adults of *C.relictus* were discovered in the natural stands in Gwangneung forest (Soribong, Mt. Jukyeop-san), while larvae and most signs of habitation were discovered from *Carpinuslaxiflora* and *Quercus* spp. in north of Mt. Soribong. Host plants with signs of habitation were located on the northern slope, especially close to the valley, where the terrain was not steep. Because the northern slope receives less sunlight and has a lower rate of moisture evaporation, the temperature is cooler, the humidity is higher, and, being close to the valley, the host plants retain moisture better; this indicates that appropriate humidity in the surrounding environment is a very important factor for habitation of *C.relictus*.

Gwangneung forest is protected as a UNESCO biosphere reserve, but it is located very close to Seoul and is becoming increasingly isolated due to road construction and the development of the surrounding land as part of the expansion of the greater capital area. Habitat disruption caused by this human interference will have a significant negative effect on maintaining a stable *C.relictus* population.

The Ussurisky nature reserve in Russia, the type locality for *C.relictus*, is located at latitude 43°33'–43°47'N, longitude 132°15'–132°47'E, and has a total area of approximately 40,432 ha, with a width of 40 km and a length of 19.5 km; this is 17 times the area of Gwangneung forest (Figure 2B). The terrain is mostly flat and low-lying, with the tallest peak at an altitude of 600 m. The mean annual temperature is 2.7 °C and the mean annual rainfall is 750–800 mm (Institute of Biology and Soil Sciences 2003).

The survey area was a flat forest dominated by Ulmusdavidianavar.japonica, which is a host plant for *C.relictus*. We observed large, old U.davidianavar.japonica trees that immature stages of *C.relictus* could inhabit, as well as trees producing sap, which is main food source for adults. The area contains many tributaries, and so, despite the relatively low rainfall, humidity is well maintained within the forest, which is thought to provide a suitable environment for *C.relictus* habitation (Fig. 13C). Moreover, like Gwangneung forest, the nature reserve is designated as a nationally protected area, making it safe from indiscriminate felling and collection, and, because it has a broad area and is located far from urban areas, it is thought to be largely unaffected by disruption of the surrounding environment.

### Host plants

There are seven known species of host plant for *Callipogonrelictus* in Korea: *Carpinuscordata*, *C.laxiflora*, *Fraxinusmandschurica*, *F.rhynchophylla*, *Quercusaliena*, *Q.mongolica*, and Ulmusdavidianavar.japonica. In our survey, we have found larvae and signs of habitation of *C.relictus* in Gwangneung forest in the following four species of tree: *Carpinuscordata*, *C.laxiflora*, *Quercusaliena*, and *Q.mongolica* (Figs 5A–D, 7B). These were most commonly discovered in *Carpinuslaxiflora*, though we also observed a large number of feeding scars in *Quercus* spp. of dead trees that could not be accurately identified at the species level, due to the high extent of decay. If these dead trees could be identified, it is possible that new host plants could be reported.

In Russia, ten host plants of *C.relictus* have been known (including *Quercusmongolica*, *Ulmuslaciniata*, Ulmusdavidianavar.japonica and *Fraxinusmandschurica*), but although we identified various host plants in the survey in the Ussurisky nature reserve, *C.relictus* emergence holes and feeding scars were only found in Ulmusdavidianavar.japonica (Figure 13B), suggesting that *C.relictus* prefer this species.

The host plants where individuals and signs of habitation of *C.relictus* were found were either dead trees that had low vigor or had died recently and were not in a state of advanced decay (Figure 13A, B). Meanwhile, given that fungi or mold infection were observed on host plants with signs of habitation (Figure 6A, B), we assume that habitation is closely related to fungi, and although there has been a study showing that fungus aid larval growth in indoor rearing (Lee et al. 2017), there has been no detailed research on the association with fungi in the natural environment.
